# Antiviral Activity of 3(2H)- and 6-Chloro-3(2H)-Isoflavenes against Highly Diverged, Neurovirulent Vaccine-Derived, Type2 Poliovirus Sewage Isolates

**DOI:** 10.1371/journal.pone.0018360

**Published:** 2011-05-25

**Authors:** Lester M. Shulman, Danit Sofer, Yossi Manor, Ella Mendelson, Jean Balanant, Anna Laura Salvati, Francis Delpeyroux, Lucia Fiore

**Affiliations:** 1 Central Virology Laboratory, Public Health Services Israel Ministry of Health, Chaim Sheba Medical Center, Tel Hashomer, Israel; 2 Department of Epidemiology and Preventive Medicine, Sackler Faculty of Medicine, University of Tel Aviv, Ramat Gan, Israel; 3 Biology of Enteric Viruses, Institut Pasteur, Paris, France; 4 Institut National de la Santé et de la Recherche Médicale (INSERM) U994, Paris, France; 5 National Center for Immunobiologicals Research and Evaluation (CRIVIB), Istituto Superiore di Sanità, Viale Regina Elena, Rome, Italy; The Scripps Research Institute, United States of America

## Abstract

**Background:**

Substituted flavanoids interfere with uncoating of Enteroviruses including Sabin-2 polio vaccine strains. However flavanoid resistant and dependent, type-2 polio vaccine strains (minimally-diverged), emerged during *in vitro* infections. Between 1998–2009, highly-diverged (8 to >15%) type-2, aVDPV_2_s, from two unrelated persistent infections were periodically isolated from Israeli sewage.

**Aim:**

To determine whether highly evolved aVDPV_2_s derived from persistent infections retained sensitivity to isoflavenes.

**Methods:**

Sabin-2 and ten aVDPV_2_ isolates from two independent Israeli sources were titered on HEp2C cells in the presence and absence of 3(2H)- Isoflavene and 6-chloro-3(2H)-Isoflavene. Neurovirulence of nine aVDPV_2_s was measured in PVR-Tg-21 transgenic mice. Differences were related to unique amino acid substitutions within capsid proteins.

**Principal Findings:**

The presence of either flavanoid inhibited viral titers of Sabin-2 and nine of ten aVDPV_2_s by one to two log_10_. The tenth aVDPV_2_, which had unique amino acid substitution distant from the isoflavene-binding pocket but clustered at the three- and five-fold axies of symmetry between capsomeres, was unaffected by both flavanoids. Genotypic neurovirulence attenuation sites in the 5′UTR and VP1 reverted in all aVDPV_2_s and all reacquired a full neurovirulent phenotype except one with amino acid substitutions flanking the VP1 site.

**Conclusion:**

Both isoflavenes worked equally well against Sabin 2 and most of the highly-diverged, Israeli, aVDPV_2_s isolates. Thus, functionality of the hydrophobic pocket may be unaffected by selective pressures exerted during persistent poliovirus infections. Amino acid substitutions at sites remote from the drug-binding pocket and adjacent to a neurovirulence attenuation site may influence flavanoid antiviral activity, and neurovirulence, respectively.

## Introduction

Poliovirus is a member of the *Picornaviridae*. Like other members of the *Picornaviridae*, poliovirus RNA is encapsidated in an icosahedral structure with axes of three-fold and five-fold symmetry formed from 60 capsomeres containing one copy each of viral capsid proteins VP1, VP2, VP3 and VP4 [1]. The binding site for the human poliovirus receptor is located in a canyon at the five-fold axis of symmetry. The VP1 of picornaviruses contain a hydrophobic pocket that is accessed through this canyon. This pocket is normally occupied by pocket factors, sphingosine-like molecules including palmitic and myristic acids and hydrophobic compounds, that stabilize the capsid and whose removal is a necessary prerequisite for uncoating [2].

Flavanoids and flavonoids were shown to have antiviral activity *in vitro* against several picornaviruses including type 2 polioviruses [3], [4], [5], [6], [7], [8]. These compounds act primarily by occupying the hydrophobic pocket, thus interfering with virus uncoating. They did not directly affect viral adsorption or RNA synthesis [4]. Mutations that interfered with the antiviral activity of these compounds appeared during *in vitro* replication of type 2 polio vaccine strains in the presence of these compound [4]. Some mutations acted directly by affecting binding while others exerted their effect indirectly by altering viral capsid stability.

Since the adoption of the Global Poliomyelitis Eradication Initiative of the WHO in 1988 [World Health Assembly resolution WHA41.28], the use of live attenuated trivalent Sabin polio vaccine, inactivated Salk polio vaccine, or a combination of both has drastically reduced the global morbidity and mortality caused by the three types of poliovirus [For latest update see http://www.polioeradication.org/]. Three problems have contributed towards the delay in achieving global eradication; failure to vaccinate all of the children in a region as in Nigeria [9], [10], [11], [12], oral vaccine failure probably related to interference by other enteric microorganisms coupled with the presence of high amounts of wild poliovirus in the environment, as in the Bihar and Uttar Pradesh States in India [11], [13], [14], and relatively rare outbreaks of poliomyelitis caused by vaccine-derived polioviruses (VDPVs) that reverted to wild phenotype [15], [16]. A VDPV is a poliovirus that has evolved from one of the three vaccine serotypes after prolonged replication, probably sustained by low immunity coverage or immunodeficiency and whose VP1 capsid protein sequence has diverged from its respective oral vaccine serotype by 1 to 15% [17]. When divergence is >15%, but an isolate can be phylogenetically linked to a previously identified VDPV, it is still designated as a VDPV. VDPVs evolve during person-to-person transmission of live-attenuated vaccine (cVDPVs) or through persistent infections of immunodeficient individuals (iVDPVs) [16]. Ambiguous VDPVs, such as environmental isolates, for which the evolutionary pathway cannot be assigned, are called aVDPVs.

Israel has been poliomyelitis free since 1989. A countrywide sewage surveillance program was introduced in 1989 to search for the presence or circulation of wild poliovirus or VDPVs before the occurrence of cases of poliomyelitis [18]. In 1998 a polio virus isolate that had diverged from Sabin 2 by 8% was identified in a sewage sample from a collection site serving a population of 1.6 million in central Israel [19]. Iterative introduction of new surveillance sites at major branch points upstream of sites yielding positive samples was used to increase the chance of isolating aVDPVs and to localize the geographical region of the excreter or excreters. Between 1998 and 2009, a total of 43 aVDPVs that had diverged from Sabin 2 vaccine by 8 to >15% were recovered from 33 sewage samples [20]. Based on time clocks for the accumulation of synonymous single nucleotide substitutions, this would correspond to a chain of continuous replication of at least 8 to 15 years in one or more human host [21]. Sequence analyses strongly suggested that all of the Israeli aVDPV_2_ isolates arose during persistent infections of immune deficient individuals, and that there were two separate epidemiological sources of persistent infections in central Israel [22]. This conclusion was strengthened when surveillance sites were introduced at upstream locations and sewage samples from a trunk line serving <350,000 individuals exclusively yielded isolates from one of the two phylogenetically related groups, while another trunk line serving 50,000 individuals exclusively yielded isolates from the other phylogenetically related group. Some of the aVDPV_2_ isolates from both groups were tested and shown to be highly neurovirulent in a transgenic mouse model system and amino acid substitutions in neutralizing antigenic epitopes resulted in three-fold lower geometric mean neutralizing antibody titers obtained against vaccine strains in sera from cohorts of Israeli subjects aged between 15 months and 50 years [22].

One potential source for reemergence of poliomyelitis after eradication is from unidentified persistent infections. When persistently infected individuals were identified (approximately 40 throughout the world to date) [15], efforts to cure the persistent infections were in most cases not successful, as exemplified by attempts to treat an immune-deficient individual in the United Kingdom [23]. However, a combination of gammaglobulin replacement therapy and pleconaril appeared effective for another persistently infected individual [24]. There is currently no universal, safe, orally administered antiviral that is effective against poliovirus. Thus there remains an urgent need to develop effective antivirals for curing persistent as well as transient poliovirus infections as a complimentary or alternative approach to oral polio vaccine for persistently infected individuals during the final stages of eradication and for post-eradication re-emergence [25], [26], [27].

The present study was undertaken to determine whether highly-diverged, Sabin 2 derived aVDPVs of different lineages that evolved *in vivo* from two different as yet unidentified epidemiological sources remained sensitive to isoflavenes *in vitro* as a first step in evaluating the potential of these compounds to reduce viral loads *in vivo* and or eliminate one or more lineage of VDPV that may co-exist [22], [28] in persistently infected immune deficient individuals.

## Materials and Methods

### Ethics Statement

This study did not require ERB approval. The viruses used in these studies were anonymous aVDPVs isolated from environmental samples. All animal model studies reported here were approved by and conducted in accordance with the guidelines of the Office of Laboratory Animal Care at the Pasteur Institute and complied with French laws and regulations. Our study was registered under the number 08188.

### Compounds

3(2H)-Isoflavene (C2) and 6-chloro-3(2H)-Isoflavene (C10) were synthesized as described [29] [25]. Stock solutions were prepared at a concentration of 1 mg per ml in ethanol and used at a final concentration of 20 µM. An equivalent volume of ethanol was added to control cultures.

### Cell lines and viruses

HEp2C cells (ATCC CCL136) were grown at 37°C in Eagles MEM supplemented with 10% fetal bovine serum (FBS), and penicillin, 160 U/ml; streptomycin, 0.32 g/ml; and mycostatin, 20 U/ml (cell medium).

Sabin 2 strains of polio vaccine were obtained from Radu Crainic, the Pasteur Institute, Paris, France and from NIBSC, Hertfordshire, England. Ten highly diverged aVDPV2s representing three lineages each from epidemiological groups 1 and 2 [22] were isolated as described from sewage samples collected from central Israel between 1998 and 2007 [22], [30] [21], [26]. Isolation of the aVDPVs included selection for growth at 40°C within 5 days. However, all isolates behaved like Sabin 2 at 37°C. High titered viral stocks were prepared by infection of HEp2C cells at 37°and all subsequent experiments were performed at this temperature. Partial genomic sequences of the aVDPV_2_ isolates reported here have been deposited in the EMBL/GenBank/DDJB database under the following access numbers: PV2_4568-1_ISR98 (SD-98-01; AM040035), PV2_5021-1_ISR99 (SD-99-01; AM040036), PV2_6056-3_ISR04 (SD-04-01; AM056049), PV2_6316-1_ISR05 (SD-05-01; AM056050), PV2_6526-1_ISR06 (SD-06-01; AM292219), PV2_6669-1_ISR06 (SD-06-05; HQ703551), PV2_6670-1_ISR06 (SD-06-06; HQ703552), PV2_6680-1_ISR06 (SD-06-09; HQ703553), PV2_6701-1_ISR06 (SD-06-10; HQ703554), and PV2_6818-1_ISR07 (SD-07-03; HQ703555). For convenience all VDPV isolate names have been shortened to “SD” for Sabin-derived, a two digit number indicating the year of isolation, and another two digits to indicate the order of isolation with in a year when there was more than one isolate (i.e. pv2-6670-1_ISR06 being the sixth isolate in 2006, has been renamed SD-06-6).

### Viral inhibition assay

Confluent monolayers of HEp2C cells were prepared in individual wells of 96-well microtiter plates by plating 60–70,000 cells per well in 100 µl of cell medium and incubating the plates overnight at 37°C in a CO_2_ incubator. C2 or C10 compounds or the equivalent volume of ethanol solvent was added to 25 µl of cell medium so that final isoflavene concentration in the wells was 20 µM. After 6 hours, cell monolayers were infected with 25 µl of a ten-fold serial dilution of virus stocks in cell medium with 2% FBS. Four replicate wells were used for each dilution. At the end of 72 hours of incubation at 37°C, cells were fixed and stained with 4% formaldehyde and crystal violet. The mean difference in TCID_50_ was determined for each virus grown in the presence or absence of isoflavenes from three independent experiments using the Karber formula.

### Neurovirulence test

Phenotypic neurovirulence was determined for nine of the ten aVDPV_2_ isolates selected for this study using PVR-Tg21transgenic mice [22], [24] [21], [23] (provided by A. Nomoto and T. Nomura). Eight-week old PVR-Tg21 transgenic mice were inoculated intranasally (i.n.; 4 males; 3 females) or intraperitoneally (i.p.; 4 males; 3 females) with 10^6^ and 10^7^ TCID_50_ infectious doses of each aVDPV isolate, respectively, and monitored daily for paresis, paralysis, or death over a 21-day interval. All inoculation experiments included fully attenuated and neurovirulent controls in order to ensure that the inoculation process by itself was never the cause of disease and that a highly neurovirulent inoculum lead to disease or death in 100% of infected animals. The titers of the virus stocks were confirmed before and after challenge.

### Genetic analysis of capsid proteins and 5′UTR

A portion of the 5′UTR and the entire P1 genomic region encoding all four capsid proteins was sequenced for all of the aVDPV_2_s used in this study using primers and RT-PCR as described in Shulman et. al [22]. Sequences were translated *in silico* and compared to that of Sabin 2 (AY184220) using the Sequencher program (Gene Codes Corp, Anne Arbor, Mi.). Genotypic reversion to neurovirulence was evaluated by determining the nucleotide at position 481 in the 5′UTR and the amino acid at position 143 in VP1. Nearest-neighbor phylogenetic analysis was performed using Clustal X [31] after bootstrapping data 1000 times and the resulting phylogenetic tree visualized using the njplot program [32]. Computer-assisted modeling of the location of substituted amino acids was performed using the Mac PyMol program (DeLano Scientfic. Palo Alto, Ca) to identify equivalent positions on the three-dimensional structural [33] of PV2 Lansing strain (PDB access number 1EAH).

### Statistical analysis

A standard analysis of variance (SPSS, v18) was used to determine the significance of differences in response to C2 and C10 between all pairwise combinations among Sabin strains and aVDPV_2_s. Both a Mixed Model for Repeated Measures (SAS v9.1) and Tukeys method for multiple comparisons of repeated measures of variants (SPSS, v18) were used to determine whether C10 treatment reduced viral titers to a greater extent than C2 for all aVDPV_2_s. Pairwise statistical comparisons between virus-associated disease development were made according to the Kaplan-Meier method and tested for significance by using the log-rank test.

## Results

### Genomic characterization of reversion to neurovirulence of aVDPV_2_ isolates used in this study

**Figure 1:**
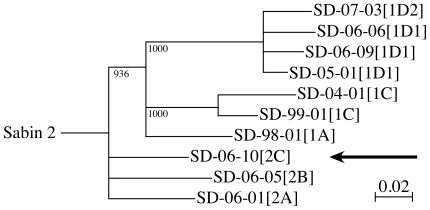
Phylogenetic relationships among type 2 aVDPVs isolated from sewage in Israel between 1998 and 2007. A nearest-neighbor phylogenetic tree was prepared from the VP1 sequences (903 nt) of type 2 aVDPVs isolated from each of the different lineages that were associated with two different epidemiological sources [22] in central Israel using Clustal X and nj plot after bootstrapping sequence data 1000 times. Branches with bootstrap values less than 900 have been collapsed. The arrow indicates the isolate that did not respond to either the C2 or C10 compounds. The numbers in brackets indicate whether the isolate belongs to group 1 or 2 and the letter or letter and number that follows indicates the lineage or sub lineage within each group.

Ten highly diverged aVDPV2 isolates representing different lineages of type 2 poliovirus from two epidemiologically unrelated sources in Israel [20], [22] were selected for analysis. A portion of the 5′UTR and the complete P1 genomic region coding for VP1, VP2, VP3 and VP4 capsid proteins were sequenced for all isolates. All ten of the aVDPV2s had reverted to neurovirulent genotypes [34], i.e., all had a reverted at nucleotide position 481 in the 5′UTR (A to G) and at amino acid residue 143 of VP1 (isoleucine encoded by ATT to threonine encoding by ACT). The phylogenetic relationship between the VP1 genes of all of the aVDPV_2_ isolates and Sabin 2 is shown in Fig. 1.

### Phenotypic characterization of reversion to neurovirulence of the aVDPV_2_ isolates

**Figure 2:**
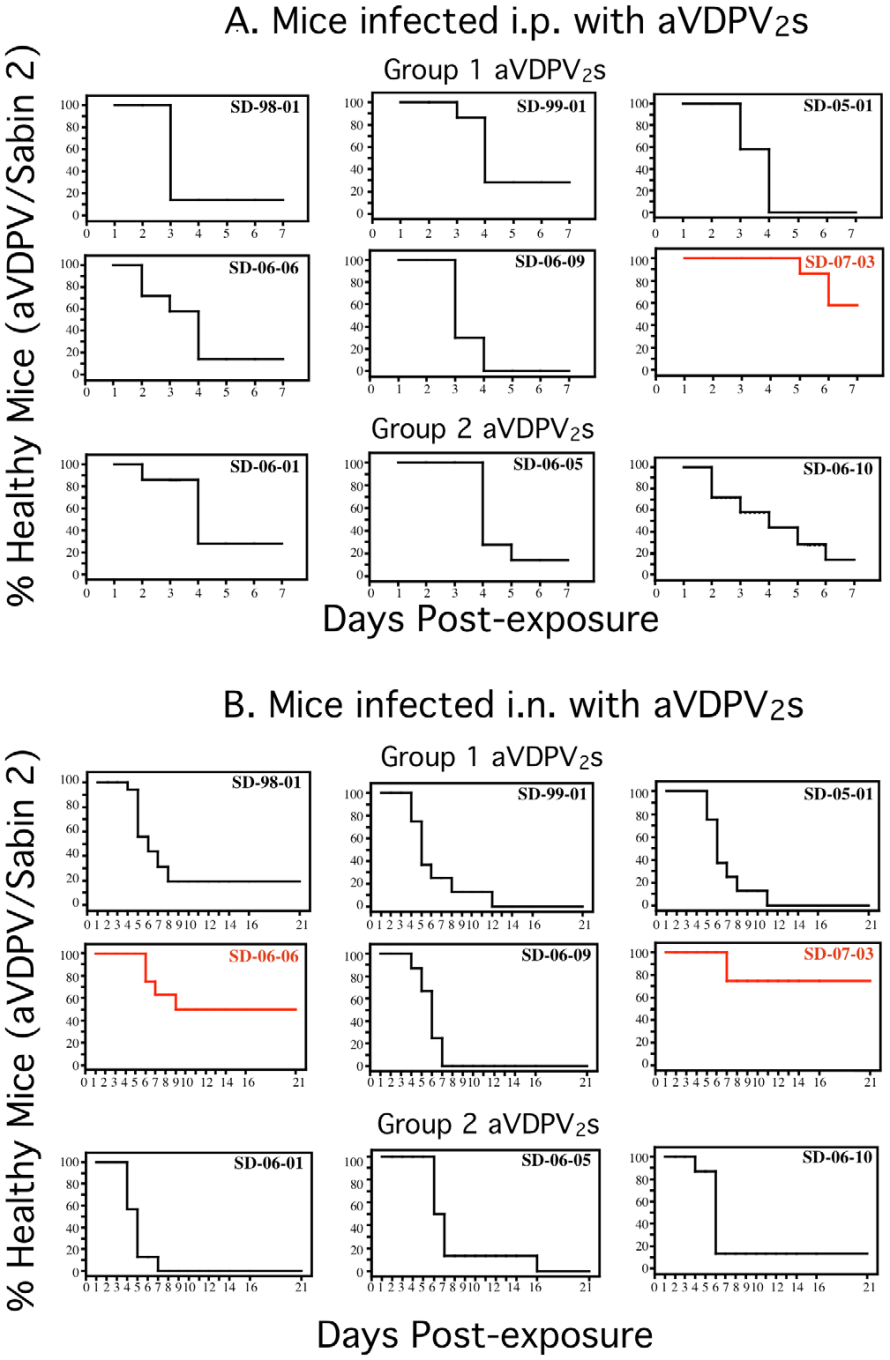
Neurovirulence of the aVDPV_2_ isolates used in this study. Neurovirulence was determined for each aVDPV_2_ isolate in PVR TG-21 mice as described in Materials and Methods. Groups of four male and four female eight week-old PVR-TG21 mice were infected i.p. with 10^7^ TCID_50_ of each aVDPV_2_, while four male and three female mice were infected i.n. with 10^6^ TCID_50_ of each aVDPV_2_. The graphs show daily percent of mice that remained healthy during seven days or 21 days following i.p. and i.n. All mice in the groups injected by either route with equivalent titers of Sabin 2 remained healthy throughout the observation periods and all mice injected with fully neurovirulent controls became sick or died (not shown). Graphs in red indicate those isolates that had not completely reverted to a neurovirulent phenotype despite the fact that all of the aVDPV2 isolates had reverted to the neurovirulent genotype, ie had G substituted for A at nucleotide 481 of their 5′UTR and threonine substituted for isoleucine at amino acid residue143 of their VP1.

Nine of he highly diverged aVDPV isolates were used to challenge PVR-Tg21 mice as described in Methods. The percentage of healthy mice, i.e. those that had not developed paresis or paralysis or had died was determined daily after i.p. or i.n. inoculation (Fig. 2). Consistent with the genotypic reversion to neurovirulence, less than 25% of the mice in groups innoculated i.p. with eight of the aVDPV_2_ isolates and less than 28% of those inoculated i.n. with seven of the eight aVDPV_2_s remained healthy after five or eight days, respectively. Unexpectedly, more than 50% of the mice inoculated with isolate SD-07-03 by either route, or SD-06-10 by the i.n. route, remained healthy at the end of the observation periods (58% at 7 days post-i.p., 75% at 21 days post-i.n., and 50% days post-i.n., respectively). Isolate SD-07-03 was significantly less neurovirulent than the eight other isolates following the i.n. and i.p. routes according to the log -rank test (p≤0.05 for 14 of 16 pairwise comparisons). Isolate SD-06-01 appears less neurovirulent in many cases (i.p. route: among 8 isolates p<0.05 in 4 cases and p<0.1 in 3 other cases).

### Inhibition of type 2 vaccine derived poliovirus (aVDPV) replication in the presence of C2 and C10 compounds

**Figure 3:**
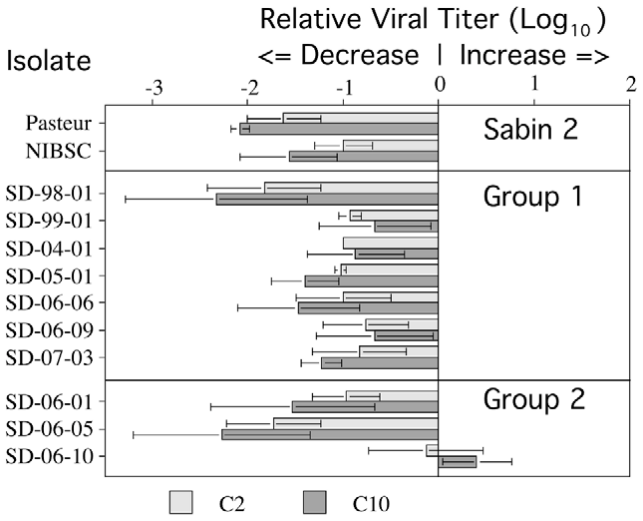
The antiviral effects of C2 and C10 compounds on aVDPV_2_ strains isolated from sewage in Israel between 1998 and 2007. The environmental isolates represent different lineages of two unrelated epidemiological events. Replicate monolayers of HEp2C cells in 96 well micro-titer plates were infected with 10-fold serial dilutions of Sabin 2 from two different sources and with each aVDPV_2_ isolate. Infection was in the absence or presence of 20 µm of the C2 or C10 compounds. The graph shows the mean of the log_10_ of the TCID_50_ from cell monolayers treated with C2 (light shading) or C10 (dark shading) minus the log_10_ of the TCID_50_ from untreated cell monolayers. The bars indicate the standard deviation from three separate experiments.

The mean difference in viral titers for type 2 Sabin (2 sources) and the 10 aVDPV_2_ polioviruses grown in the presence and absence of C2 and C10 compounds was determined from three independent titrations (Fig. 3). Standard analysis of variance (SPSS) revealed that isolate SD-06-10 from epidemiological Group 2, was the only aVDPV_2_ isolate that had a significantly different response than the Sabin 2 Pasteur strain in the presence of either compound (p<0.01) and Sabin 2 NIBSC strain in the presence of C10 (p<0.05). Furthermore SD-06-10 was also significantly different (p<0.05) in its response to C10 from 5 of the other 9 aVDPV_2_s (the exceptions were SD-99-01, SD-06-09, SD-04-01 and SD-07-03; all from epidemiological Group 1). The replication of aVDPV_2_ isolate SD-06-10 was unaffected when grown in the presence of either compound. The change in SD-06-10 associated with C10 was not statistically different from 0; the variation was too large to exclude the possibility that the average increase (even though each sample increased) was due to chance. For the Sabin 2 reference strains (from two different sources) and the other nine aVDPV_2_s (isolated from two unrelated epidemiological events), the presence of either compound reduced viral growth by one to two logs. The mean differences in the presence of the drugs and their standard errors are as follows: C2 −1.07, SE = 0.07; C10: −1.31, SE = 0.10. A repeated measures analysis of variance (Tukey's Method, SPSS), which takes into account the paired nature of the C2/C10 differences, revealed that these mean differences were statistically different (p = 0.003). However, the C2 vs C10 comparison was not consistent across all isolates, as there was a significant interaction (p = 0.038). These findings are consistent with results from analysis using the Mixed Model for Repeated measures of the SAS (v9.1) program that also indicated that the C10 compound was significantly more effective than the C2 compound in reducing viral titers for the nine aVDPVs that responded to the antiviral affects of the two isoflavenes (t value 30.26; P<0.0001).

### Three-dimensional mapping of unique amino acid substitutions in capsid proteins of the isoflavene-resistant aVDPV isolate, SD-06-10, and the neurovirulence-attenuated aVDPV isolates, SD-07-03

C2 and C10 compounds exert their antiviral effect by interacting with amino acids in the viral capsid pockets. Since amino acid substitutions might have direct or indirect effects on this interaction, the P1 sequences from the previous section were translated *in silico* using the Sequencher program to determine which amino acid substitutions in the capsid proteins were unique in the SD-06-10 strain or shared with the other aVDPV_2_ isolates. The unique and shared substitutions are listed in Table S1. Also included in the table are the amino acid residues that define the hydrophobic pocket as well as the substitutions conferring isoflavene resistance or dependence to Sabin 2 as reported by Salvati et al. [4].

**Figure 4:**
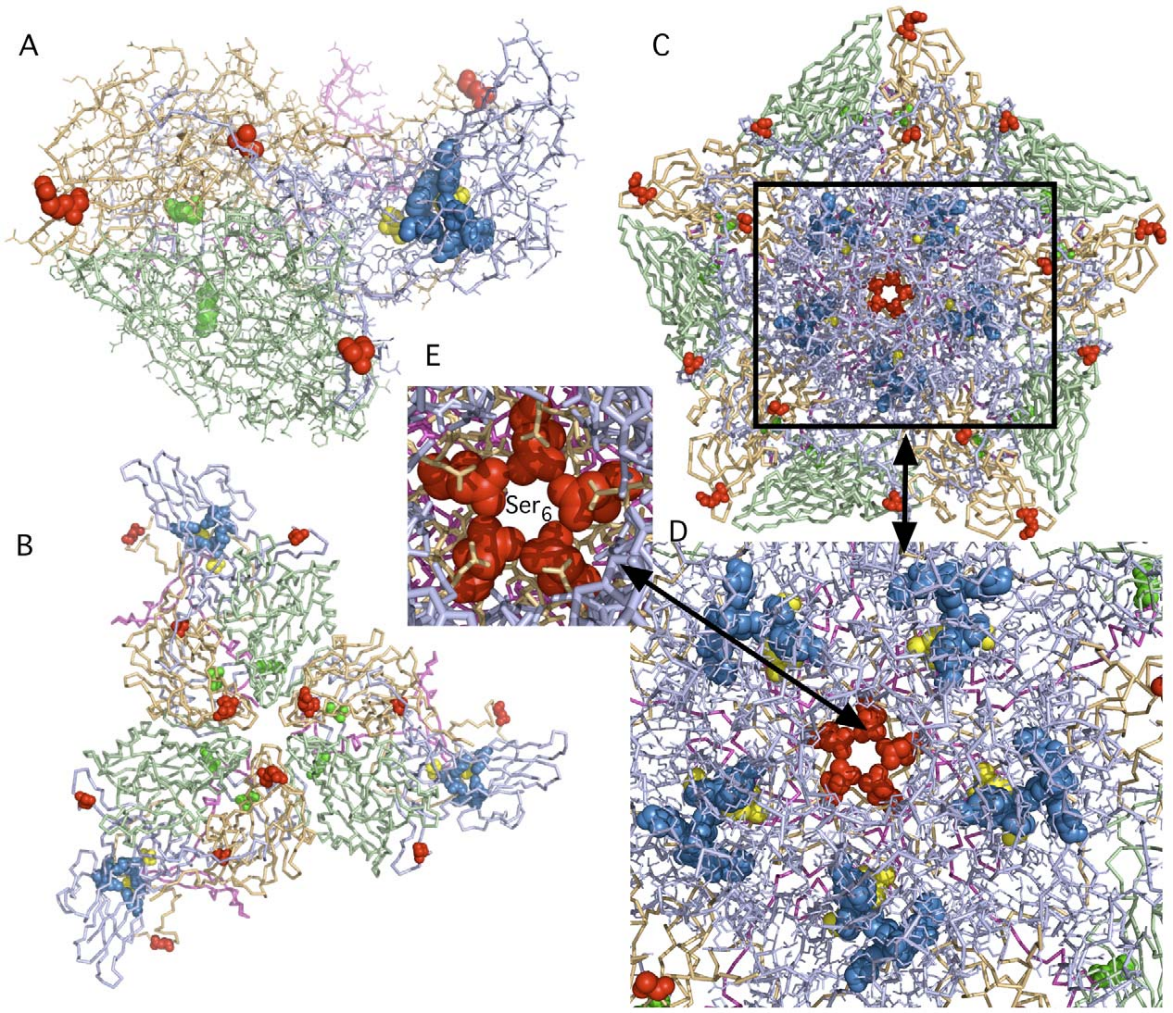
Unique amino acid substitutions in the capsid proteins of an isoflavene resistant aVDPV2 isolate SD-06-10 mapped onto the three-dimensional x-ray crystallographic atomic coordinates of type 2 poliovirus (PDB accession number1EAH). aVDPV_2_ isolate SD-06-10 was resistant to the antiviral effects of both C2 and C10 compounds (Fig. 3) The *in silico* translated amino acid sequences of the capsid proteins of all of the aVDPV2 isolates and Sabin 2 (AY184220) were compared and amino acid substitutions in capsid proteins that were unique to SD-06-10 were determined (Table S1). The positions of these unique substitutions were mapped onto the coordinates of type 2 poliovirus (1EAH) using MacPyMol and are represented by red spheres. The hydrophobic pocket is represented by blue spheres and the position of amino-acid substitution that were previously shown to result in loss of sensitivity to or dependence on isoflavenes are indicated by the yellow and green spheres, respectively. (**A**) A line model of a single capsomere containing one copy of VP1 (light blue), VP2 (pale green), VP3 (light orange) and VP4 (light magenta). (**B**) A stick model representing three capsomeres at the three-fold axis of symmetry. (**C**) A stick model representing five capsomeres at the five-fold axis of symmetry. (**D**) An enlargement of the central portion of figure (D) showing the relation between the five-fold axis and the hydrophobic pockets (**E**) A further enlargement showing the position of Ser_6_ of VP3 at the center of the five-fold axis. All views are from an external viewpoint. An animated “Interactive 3D Complement” (I3DC) for the structures in this figure appears in *Proteopedia* at http://proteopedia.org/w/Journal:PLoS_ONE:1.

The unique amino acid substitutions in isolate SD-06-10 were mapped onto the three-dimensional x-ray crystallographic atomic coordinates of type 2 poliovirus (PDB accession number1EAH) (Fig. 4) using MacPyMol to determine their location with respect to the isoflavene-binding pocket and to the capsomere junctions. None of the unique substitutions appear to directly interact with the binding pocket or with amino acid substitutions that were previously identified by Salvati et al. [4] as those that indirectly affected the response to isoflavenes. However, one unique substitution at amino acid residue 6 of VP3, Ser_6_, was located in the central junction of the five capsomeres at the five-fold axis of symmetry (Fig. 4E.) and another unique substitution at residue 206 of VP3, Ser_206_, was located adjacent to the three fold axis of symmetry (Fig. 4B) where they might influence capsid flexibility and or stability Among all of the aVDPV isolates that were sensitive to C2 and C10 and resistant strain SD-06-10, only isolate SD-04-01, had two amino acid substitutions closer than five residues from any of the amino acids listed in part a of Table S1 and highlighted in blue for Fig. 4 that represent the position of the hydrophobic pocket. One was adjacent to VP1 residue 110 and the other occurred two residues after VP1 residue 136.

**Figure 5:**
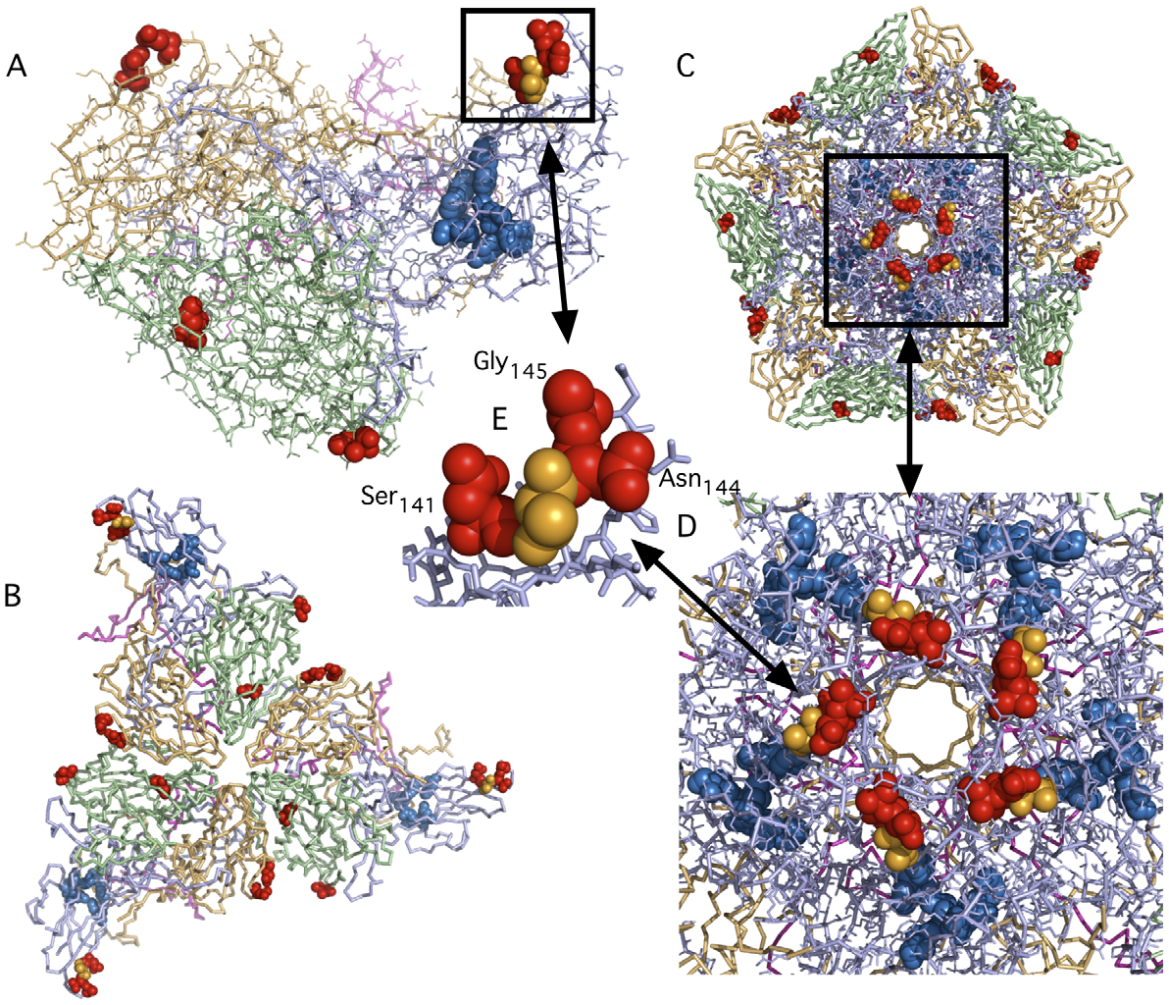
Unique amino acid substitutions in the capsid proteins of SD-07-03, an aVDPV_2_ isolate that reverted genotypically but not phenotypically to neurovirulence, mapped onto the three-dimensional x-ray crystallographic atomic coordinates of type 2 poliovirus (PDB accession number1EAH). The positions of unique substitutions of SD-07-03 (Table S1), determined as in Fig. 4, were mapped onto the coordinates of type 2 poliovirus (1EAH) using MacPyMol and are represented by red spheres. The hydrophobic pocket is represented by blue spheres and the position of amino acid 143 of VP1, one of two determinants for neurovirulence in type 2 poliovirus, is represented by orange spheres. (**A**) A line model of a single capsomere containing one copy of VP1 (light blue), VP2 (pale green), VP3 (light orange) and VP4 (light magenta). (**B**) A stick model representing three capsomeres at the three-fold axis of symmetry. (**C**) A stick model representing five capsomeres at the five-fold axis of symmetry. (**D**) An enlargement of the central portion of figure (D) showing the relation between the five-fold axis and the hydrophobic pockets (**E**) An enlargement showing the position of substituted amino acids Serine_141_ Asparagine_144_ and Glycine of VP1 (red spheres) relative amino acid Threonine_143_ (orange spheres) of VP1. All views are from an external viewpoint. An animated “Interactive 3D Complement” (I3DC) for the structures in this figure appears in *Proteopedia* at http://proteopedia.org/w/Journal:PLoS_ONE:1.

A substitution of one or more of the nucleotides encoding amino acid 143 of VP1 that results in replacement of Ile_143_ with Thr_143_ is one of the two mutations considered necessary and sufficient to reverse attenuation of neurovirulence for Sabin 2. The other is the substitution of A with a G at nt position 481 of the 5′UTR. Since both substitutions were present in the genomes of all ten of the aVDPV2s, all should have possessed a neurovirulent phenotype. One isolate, SD-07-03, however, exhibited an almost fully attenuated phenotype in transgenic mice infected by both the i.p. and i.n. routes despite being derived from highly nurovirulent ancestors and having both substitutions. Unique and shared amino acid substitutions in the capsid proteins for isolate SD-07-03, listed in Table S1, were mapped onto the 3-D capsid structure to infer whether any of them might have contributed towards this attenuated phenotype. Unique substitutions found in this strain at residues 141, 143, and 145 of VP1 that flank Thr_143_ (Fig. 5E) were more likely to counteract the effect of the reversion at residue 143 than substitutions in the 5′ and 3′ untranslated regions or in the P2 and P3 coding regions (not sequenced) although the contribution of substitutions in the non-capsid regions of the genome can not be ruled out. All three substitutions are also located adjacent to the 5-fold axis of symmetry. None of SD-07-03's unique substitutions were located near the 3-fold axis of symmetry.

An animated “Interactive 3D Complement” (I3DC) for the structures in Fig. 4 and Fig. 5. appears in *Proteopedia* at http://proteopedia.org/w/Journal:PLoS_ONE:1.

## Discussion

Permanent global eradication of poliomyelitis requires the elimination of all chains of transmission of poliovirus throughout the world through immunization programs and improvement of hygienic conditions and the subsequent containment and destruction of all stocks of poliovirus. Most national immunization programs are based on the use of live oral Sabin vaccines (OPV) or a combination of OPV and inactivated Salk vaccines (IPV). Of the three impediments for successful eradication, failure to vaccinate and vaccine failure result in continued person-to-person transmission of wild polio viruses and VDPVs. Intense efforts are underway to increase vaccine coverage with conventional vaccines to overcome the first problem, and to develop new vaccines or vaccination strategies to deal with the second problem [11]. It is of critical importance to rapidly reach and maintain effective high coverage levels in order to provide herd immunity and prevent outbreaks of poliomyelitis by person-to-person transmission of minimally diverged OPV and VDPV as is currently occurring in Nigeria [12], [35]. The third problem, continuous or intermittent excretion by anonymous [36] or immune deficient individuals [12], [37] persistently infected after exposure to polio vaccine (or wild poliovirus) is more complex and has two facets that must be dealt with.

The first is to determine the number of epidemiological foci of persistent infections and identify the responsible individuals [12], [36], [38]. To date, less than 40 individuals throughout the world have been identified as individuals who excrete iVDPVs as a consequence of persistent poliovirus infections. These individuals had complete or almost complete B-cell immune deficiencies. Some individuals were identified after they developed symptoms of poliovirus infection. Of the remaining individuals who were identified by chance, some remained asymptomatic while others eventually developed symptoms of poliovirus infection. Currently, the number of known individuals with persistent infections has fallen since persistent infection in some individuals cleared spontaneously while others are no longer alive. However, the total number of persistently infected individuals throughout the world is unknown. Surveys of individuals with B-cell immune deficiencies for persistent poliovirus infection have so far failed to identify additional persistently infected individuals [16]. On the other hand additional persistently infected individuals must exist since increasing numbers of aVDPVs have been isolated from sewage samples as more national environmental poliovirus surveillance programs incorporate environmental surveillance [12]. Epidemiologically related aVDPVs, like the type 2 s in Israel [12], [19], [20], the type 3 s in Estonia [12], [39], and the types 1, 2, and 3 in Finland [40] that were intermittently isolated over many years are highly diverged, neurovirulent polioviruses. Unfortunately many countries do not conduct environmental surveillance, and in those that do, the probability of identifying anonymous persistent excretors is very low. Thus the global burden of such strains is unknown.

The second facet of persistent infections is to cure the subjects affected in order to prevent pre-eradication emergence or post-eradication re-emergence of foci of poliomyelitis due to person-to-person transmission of these polioviruses. It may be very difficult to identify the persistent excretor who triggers a post-eradication re-emergence after cessation of OPV immunization. However, responding with reintroduction of vaccination with live polio vaccine strains by itself might lead to further outbreaks [35], [41]. Effective antivirals may offer an alternative community response [42]. Efforts to cure one immunodeficient individual with currently available drugs were not effective [23]. To date there are no approved antiviral drugs for treating poliovirus infections although gammaglobulin replacement therapy combined with pleconaril was reported to have eliminated persistence in another individual [24]. Potential anti-viral drug targets include the 3 C protease, proteins essential for viral replication, and the capsid proteins [43]. Isoflavenes are among the group of capsid inhibitors that intercalate into the hydrophobic pocket of type 2 poliovirus capsid blocking uncoating and release of viral RNA from capsid into the cell [4], [5]. Mutations conferring resistance or dependence were either already present or arose rapidly during *in vitro* replication of polio type 2 vaccine in the presence of drugs [4]. The majority of the isolates were sensitive to isoflavenes. Isolates that developed resistance or dependence were only a minor component of the quasispecies pool and were identified after plaque selection.

More than one lineage of poliovirus may co-exist in persistently infected individuals and each lineage is present as a quasispecies. Multiple lineages were described in at least one persistently infected, immunodeficient patient [22], [28] and inferred [21] from molecular analyses of aVDPVs recovered from the two unidentified, persistently infected individuals in Israel. In this report, we demonstrated that the majority of the viruses in the quasispecies obtained after amplification of at least nine of ten highly diverged type 2 neurovirulent aVDPV_2_ plaque isolates recovered from sewage from these two anonymous sources were still as sensitive *in vitro* as Sabin 2 vaccine strains to the *in vitro* antiviral effects of C-2 and C-10 compounds at concentrations of 20 µM. The remaining isolate, SD-06-10, was unaffected by the antiviral compounds. It did not have any unique amino acid substitutions in the hydrophobic pocket that might have blocked the binding of C2 or C10. Instead, unique amino acid substitutions were observed far from the drug-binding pocket near the three-fold and at the five-fold axes of symmetry between capsomeres. Substitution of amino acids at these sites might alter capsid stability (or pocket hindrance) and block the access and binding of the C2 or C10 compounds even though they are located at sites remote from the hydrophobic pocket as observed by Salvati et al. [4].

All ten of the aVDPV_2_s were genotypically reverted to neurovirulence [34], i.e., all had reverted at nucleotide position 481 in the 5′UTR (A to G) and amino acid residue 143 of VP1 (isoleucine encoded by ATT to threonine encoded by ACT). Phenotypic reversion for nine of the ten aVDPV_2_s was determined using the murine transgenic (PVR TG21) neurovirulence test. Two different routes of inoculation, i.p. and i.n. were used and the PVR TG21 mice were observed daily for clinical signs of paresis and paralysis, or death. Seven of the genotypically reverted strains were also phenotypically neurovirulent by both routes of inoculation. Strain, SD-06-10, which was resistant to the C2 and C10 antiviral compounds, was neurovirulent when introduced i.p. but only partially neurovirulent when introduced i.n. Unexpectedly, strain, SD-07-03, which was sensitive to both antiviral compounds, was only weakly neurovirulent when administered either by the i.n. or i.p. route. These results suggest that nucleotide changes in addition to the two “classic” reversions can modulate the neurovirulence of poliovirus strains. The VP1 of SD-07-03 had diverged from Sabin 2 (AY194220) by 14.4%. Four nucleotide substitutions resulted in three amino acid substitutions in VP1 at residues 141, 144 and 145. These changes (Fig. 5E), Asn_141_, Asp_144_ and Ala_145_ to Ser_141_, Asn_144_ and Gly_145_, respectively, presumably modulated the phenotypic reversion of a genome with the “classic” neurovirulent genotype for type 2 poliovirus. However, additional substitutions in capsid proteins and or in the non-structural proteins or non-coding regions may also have contributed to the phenotype. The accumulation of substitutions in these highly diverged aVDPVs is due to natural genetic drift and digestive tract selective pressure. Relationship between this genetic drift and neurovirulence is indirect. This can lead to variants that are more or less neurovirulent in mice. In the case of the isolates described in this report, all had lost the fully attenuated phenotype of the Sabin 2 strain.

In conclusion, both isoflavenes worked equally well against Sabin 2 and most of the highly-diverged aVDPV_2_s isolated in Israel. Thus, functionality of the hydrophobic pocket may be unaffected by mutations that accumulate during selective pressures exerted during persistent poliovirus infections. The isoflavenes reduced the poliovirus loads *in vitro*. These compounds are not toxic when administered *in vivo* to mice following different routes of inoculation and at very high concentration (400 mg/kg) (L.Fiore, unpublished data). Experiments to evaluate the toxicity of the compounds on transgenic PVR Tg-21 mice and experiments to test whether isoflavenes inhibit replication *in vivo* in these mice are currently underway as first step to determine whether it may be possible to use these compounds in humans. Finally, it is worth mentioning that amino acid substitutions at sites remote from the drug-binding pocket, and others related to known neurovirulence attenuation sites may influence the antiviral activity of isoflavenes and the neurovirulence of the strains.

## Supporting Information

Table S1 **Unique amino acid substitutions in capsid proteins of aVDPV2 isolates SD-06-10 and SD-07-03.**
(DOCX)
