# Supplementary material for: Antiviral Activity of 3(2H)- and 6-Chloro-3(2H)-Isoflavenes against Highly Diverged, Neurovirulent Vaccine-Derived, Type2 Poliovirus Sewage Isolates
Source: PLoS One. 2011 May 25;6(5):e18360. doi: 10.1371/journal.pone.0018360 (PMC3102060; doi:10.1371/journal.pone.0018360)
Supplement: Table S1 — Unique amino acid substitutions in capsid proteins of aVDPV2 isolates SD-06-10 and SD-07-03. (DOCX) [file pone.0018360.s001.docx]

**Supplement Table S1. Unique amino acid substitutions in capsid proteins of aVDPV2 isolates SD-06-10 and SD-07-03.**

| **Structural Feature or Phenotype** | **Isolate** | **Capsid Protein** | **Amino Acid Residue** | **Amino Acid Substitution** | **Also in isolates** | **Figure** | **Highlite color** |
| --- | --- | --- | --- | --- | --- | --- | --- |
| **A. Hydrophobic Pocket** | |  |  |  |  |  |  |
|  | **Sabin 2** |  |  |  |  |  |  |
|  |  | VP1 | 110 | Ile | ***Prototype*** | Fig 4, 5 | ***blue*** |
|  |  | VP1 | 134 | Phe | ***Prototype*** | Fig 4, 5 | ***blue*** |
|  |  | VP1 | 136 | Phe | ***Prototype*** | Fig 4, 5 | ***blue*** |
|  |  | VP1 | 159 | Tyr | ***Prototype*** | Fig 4, 5 | ***blue*** |
|  |  | VP1 | 181 | Pro | ***Prototype*** | Fig 4, 5 | ***blue*** |
|  |  | VP1 | 194 | Ile | ***Prototype*** | Fig 4, 5 | ***yellow*** |
|  |  | VP1 | 196 | Val | ***Prototype*** | Fig 4, 5 | ***blue*** |
|  |  | VP1 | 240 | Leu | ***Prototype*** | Fig 4, 5 | ***blue*** |
| **B. Isolates resistant to isoflavenes** | | |  |  |  |  |  |
|  | **R1/01a** |  |  |  |  |  |  |
|  |  | VP1 | 131 | Asp to Val | none | Fig 4, 5 | ***yellow*** |
|  | **R2-5/01a** |  |  |  |  |  |  |
|  |  | VP1 | 194 | Ile to Met | none | Fig 4, 5 | ***yellow*** |
|  | **SD-06-10** |  |  |  |  |  |  |
|  |  | VP1 | 5 | Met to Ile | ***unique*** [Val 1-7; Lys 8] | Fig 4 | ***red*** |
|  |  | VP1 | 222 | Ser to Thr | ***unique*** [Pro 2,3,6,8,9] | Fig 4 | ***red*** |
|  |  | VP3 | 6 | Asn to Ser | ***unique*** | Fig 4 | ***red*** |
|  |  | VP3 | 96 | Ala to Ser | ***unique*** | Fig 4 | ***red*** |
|  |  | VP3 | 206 | Arg to Ser | ***unique*** | Fig 4 | ***red*** |
|  |  | VP3 | 237 | Pro to Arg | ***unique*** | Fig 4 | ***red*** |
|  |  | VP1 | 10 | Val to Ile | 1-8 | Fig 4 | none |
|  |  | VP1 | 31 | Lys to Arg | 8 | Fig 4 | none |
|  |  | VP1 | 103 | Arg to Lys | 1,2,3,6,9 | Fig 4 | none |
|  |  | VP1 | 143 | Ile to Thr | 1-9 | Fig 4 | none |
|  |  | VP1 | 171 | Asn to Asp | 1-9 | Fig 4 | none |
|  |  | VP1 | 257 | Ile to Val | 2,3,5,7 | Fig 4 | none |
|  |  | VP1 | 294 | Pro to Ser | 4-7 | Fig 4 | none |
|  |  | VP2 | 45 | Thr to Ala | 1-7 | Fig 4 | none |
|  |  | VP2 | 172 | Asn to Lys | 1-4 [Glu 5-7] | Fig 4 | none |
|  |  | VP3 | 19 | Tyr to HIis | 9 | Fig 4 | none |
|  |  | VP3 | 41 | Arg to His | 1-7,9 | Fig 4 | none |
|  |  | VP3 | 75 | Thr to Ser | 1,4-8 [Ala 2,3,9] | Fig 4 | none |
|  |  | VP3 | 78 | Ser to Thr | 1-7,9 | Fig 4 | none |
|  |  | VP3 | 197 | Arg to Lys | 1,4-8 | Fig 4 | none |
|  |  | VP4 | 42 | Ser to Asn | 1-3,9 | Fig 4 | none |
| **C. Isolates dependent on isoflavenes** | | | |  |  |  |  |
|  | **D7/01a** |  |  |  |  |  |  |
|  |  | VP4 | 58 | Lys to Glu | none | Fig 4, 5 | ***green*** |
|  | **D6/07a** |  |  |  |  |  |  |
|  |  | VP1 | 53 | Asn to Ser | none | Fig 4, 5 | ***green*** |
| **D. Isolates with attenuated neurovirulence** | | | |  |  |  |  |
|  | **Sabin 2** |  |  |  |  |  |  |
|  |  | 5'UTR | (nt 481) | A to G | all | n.s. | none |
|  |  | VP1 | 143 | Ile to Thr | all | Fig 5 | ***orange*** |
|  | **SD-07-03** |  |  |  |  |  |  |
|  |  | VP1 | 3 | Val to Met | ***unique*** | Fig 5 | ***red*** |
|  |  | VP1 | 141 | Asn to Ser | ***unique*** | Fig 5 | ***red*** |
|  |  | VP1 | 144 | Asp to Asn | ***unique*** [Glu 8] | Fig 5 | ***red*** |
|  |  | VP1 | 145 | Ala to Gly | ***unique*** | Fig 5 | ***red*** |
|  |  | VP2 | 248 | Ile to Val | ***unique*** | Fig 5 | ***red*** |
|  |  | VP2 | 265 | Ile to Val | ***unique*** | Fig 5 | ***red*** |
|  |  | VP3 | 140 | Glu to Val | ***unique*** | Fig 5 | ***red*** |
|  |  | VP3 | 147 | Glu to Asp | ***unique*** | Fig 5 | ***red*** |
|  |  | VP2 | 135 | Ala to Ser | ***unique*** | Fig 5 | none |
|  |  | VP3 | 154 | Val to Met | ***unique*** | Fig 5 | none |
|  | **SD-07-03 AND SD-06-06** | | |  |  |  |  |
|  |  | VP1 | 5 | Met to Val | 1-,6 [Lys 8; Ile 10] | Fig 5 | none |
|  |  | VP1 | 10 | Val to Ile | 1-6,8,10 | Fig 5 | none |
|  |  | VP1 | 13 | Ile to Val | 4-6 | Fig 5 | none |
|  |  | VP1 | 21 | Pro to Leu | 1-6,9 | Fig 5 | none |
|  |  | VP1 | 25 | Asn to Ser | 1-6,9 | Fig 5 | none |
|  |  | VP1 | 99 | Lys to Asn | 4-6 [Gln 1; Arg 3] | Fig 5 | none |
|  |  | VP1 | 143 | Ile to Thr | 1-6,8-10 | Fig 5 | none |
|  |  | VP1 | 171 | Asn to Asp | 1-6,8-10 | Fig 5 | none |
|  |  | VP1 | 186 | Thr to Ile | 4-6 | Fig 5 | none |
|  |  | VP1 | 218 | Ala to Glu | 1-6 | Fig 5 | none |
|  |  | VP1 | 257 | Ile to Val | 2,3,5,10 | Fig 5 | none |
|  |  | VP1 | 276 | Ala to Val | 2-6 | Fig 5 | none |
|  |  | VP1 | 280 | Phe to Tyr | 1-6,9 | Fig 5 | none |
|  |  | VP1 | 294 | Pro to Ser | 4-6,10 | Fig 5 | none |
|  |  | VP2 | 33 | Val to Ile | 5-6 | Fig 5 | none |
|  |  | VP2 | 45 | Thr to Ala | 1-6,10 | Fig 5 | none |
|  |  | VP2 | 60 | Ala to Thr | 1-6 | Fig 5 | none |
|  |  | VP2 | 155 | Glu to Val | 1,4-6,8 | Fig 5 | none |
|  |  | VP2 | 164 | Thr to Asn | 1-6 | Fig 5 | none |
|  |  | VP2 | 172 | Asn to Glu | 5,6 [Lys 1-4,10] | Fig 5 | none |
|  |  | VP2 | 186 | Val to Ala | 1-6,8 | Fig 5 | none |
|  |  | VP3 | 41 | Thr to His | 1-6,9-10 | Fig 5 | none |
|  |  | VP3 | 61 | Arg to His | 4-6 [Cys 2-3,8-9] | Fig 5 | none |
|  |  | VP3 | 75 | Thr to Ser | 1,4-6,8,10 [Ala 2-3,9] | Fig 5 | none |
|  |  | VP3 | 77 | His to Asn | 4-6 | Fig 5 | none |
|  |  | VP3 | 78 | Ser to Thr | 1-6,9-10 | Fig 5 | none |
|  |  | VP3 | 85 | Leu to Phe | 4-6 | Fig 5 | none |
|  |  | VP3 | 103 | Ile to Val | 4-6 | Fig 5 | none |
|  |  | VP3 | 175 | Thr to Ala | 2-6 | Fig 5 | none |
|  |  | VP3 | 197 | Arg to Lys | 1,4-6,8-10 | Fig 5 | none |
|  |  | VP3 | 209 | Asp to Val | 2-6,8-9 | Fig 5 | none |
|  | **SD-07-03 but NOT SD-06-06** | | |  |  |  |  |
|  |  | VP2 | 72 | Arg to Asn | 1,6 [Gly 3; Lys 4-5] | Fig 5 | none |
|  |  | VP3 | 80 | Thr to Asn | 9 [Asp 4-6] | Fig 5 | none |
|  |  | VP3 | 234 | Glu to Lys | 1-3,6,9 | Fig 5 | none |

^a^ From reference 4: Salvati et. al ., Antimicrob Agents Chemother 48:2233-43.

^b^ Numbers refer to aVDPV isolates: 1. SD-98-01(AM040035), 2. SD-99-01(AM040036), 3. SD-04-01 (AM056049), 4. SD-05-01 (AM056050), 5. SD-06-06 (HQ703552), 6. SD-06-09 (HQ703553), 7. SD-07-03 (HQ703555), 8. SD-06-01 (AM292219), 9. SD-06-05 (HQ703551), and 10. SD-06-10 (HQ703554). aVPDV isolates with different amino acid substitutions at the given residue are indicated by the substituted amino acid followed by the isolate number(s).
